# Reply to comment on “Total IgE levels in patients with hematologic malignancies”

**DOI:** 10.1016/j.waojou.2025.101122

**Published:** 2025-09-26

**Authors:** Parisa Amjadi, Fatemehsadat Hosseini, Ehsan Zaboli, Mohammad Eslami-Jouybari, Hossein Asgarian-Omran, Akbar Hedayatizadeh-Omran, Versa Omrani-Nava, Reza Alizadeh-Navaei

**Affiliations:** a Gastrointestinal Cancer Research Center, Non-communicable Diseases Institute, Mazandaran University of Medical Sciences, Sari, Iran; b Department of Immunology, School of Medicine, Mazandaran University of Medical Sciences, Sari, Iran

We appreciate the interest and comments from researchers regarding our article on “Total IgE levels in patients with hematologic malignancies”.^1^ The main concern of this letter pertains to the IgE range, and studies indicate that IgE levels are influenced by the age of subjects, community or ethnicity,^2^ and the specific assay and kit range used,^3^ which contribute to inter-individual variability. In the present study, all individuals were within the age range of adults, and children were not included. Furthermore, the comparison was not only qualitative regarding the ranges mentioned but also included quantitative comparisons and central index comparisons, in which a significant difference was observed. Additionally, there was a control group, which helped eliminate the effects of differences between communities, and the quantitative values showed a significant difference between the case and control groups, both drawn from the same population. The IgE sampling occurred on the day of receiving the first dose of chemotherapy and prior to the administration of the chemotherapeutic drug. Concerning the varying IgE levels between tumors, as noted in our initial study, IgE levels differ among various hematological malignancies and even between solid tumors and hematological malignancies.^1^ Therefore, larger, mechanistic, and prospective studies are essential to unravel these complex associations.

## Funding

Not applicable.

## Availability of data and materials

Not applicable.

## Author contributions

RAN composed the response. All co-authors reviewed and approved the text.

## Ethics approval

Not applicable.

## Consent for publication

All authors have consented to the publication of the current manuscript content.

## Declaration of competing interest

The authors have no relevant financial or non-financial interests to disclose.
